# Therapeutic Window for Intravenous Human Muse Cell Administration in Mouse Spinal Cord Injury

**DOI:** 10.3390/ijms27146219

**Published:** 2026-07-12

**Authors:** Kotaro Sakashita, Yoshihiro Kushida, Shohei Wakao, Hiroshi Takahashi, Yasuhiro Horibata, Shun Okuwaki, Yosuke Ogata, Takane Nakagawa, Takahiro Sunami, Hisanori Gamada, Tomoaki Shimizu, Toru Funayama, Kousei Miura, Hiroshi Noguchi, Hiroyuki Sugimoto, Masashi Yamazaki, Mari Dezawa, Masao Koda

**Affiliations:** 1 Department of Orthopaedic Surgery, Institute of Medicine, University of Tsukuba, Tsukuba 305-8577, Ibaraki, Japan; 2 Department of Stem Cell Biology and Histology, Tohoku University Graduate School of Medicine, Sendai 980-8575, Miyagi, Japan; 3 Department of Biochemistry, Dokkyo Medical University School of Medicine, Mibu 321-0293, Tochigi, Japan

**Keywords:** Muse cells, spinal cord injury, intravenous administration, therapeutic window, mesenchymal stromal cells, sphingosine-1-phosphate

## Abstract

Stage-specific embryonic antigen-3-positive pluripotent-like/macrophage-like multilineage-differentiating stress-enduring (Muse) cells are a distinct subpopulation of mesenchymal stromal cells (MSCs), accounting for 1% to several percent of MSCs. Although stem cell therapy for spinal cord injury (SCI) typically targets the subacute phase to avoid the hostile acute environment, the therapeutic window for Muse cells remains unclear. C57BL/6J mice with severe T9 contusion SCI received a single tail vein injection of human bone marrow-derived (BM) Muse cells, BM-MSCs (both 5 × 10^4^ cells), or vehicle at 2, 8, 14, or 28 days post-injury (DPI) without immunosuppressants. Among the different administration time points, the 2-DPI Muse cell group exhibited significantly higher Basso Mouse Scale scores than the BM-MSC and vehicle groups from 14 days after injection, while no significant differences were observed at the other administration time points. The 2-DPI Muse cell group showed significantly greater homing to the injured spinal cord than the BM-MSC group, with persistent engraftment and neural-lineage marker expression at day 42. Ablation of engrafted Muse cells at day 42 partially reversed locomotor recovery, suggesting that engrafted Muse cells contributed to functional recovery. These findings suggest that intravenous Muse cell therapy exerts timing-dependent therapeutic effects after SCI, with greater efficacy during the early post-injury phase.

## 1. Introduction

Spinal cord injury (SCI) is a life-altering condition with limited current treatments [1,2]. Although stem-cell-based regenerative medicine has emerged as a promising strategy, effective therapies have not yet been established [3]. Maximizing therapeutic efficacy requires elucidating the optimal therapeutic window [4]. Because SCI biology evolves from an inflammation-dominated acute phase to a glial scar-dominated chronic phase, engraftment challenges differ across stages [5]. Preclinical studies suggest that for neural progenitor/stem cells (NPC/NSCs)-based approaches, transplantation during the subacute phase yields the most robust functional recovery [6,7]. This timing avoids the hostile inflammatory milieu of the acute phase, in which surviving grafted cells tend to differentiate preferentially into glial rather than neuronal lineages, while preceding the completion of glial scar formation [8,9]. In contrast, mesenchymal stromal cells (MSCs) exert their effects primarily through paracrine mechanisms [10]. While acute phase administration of MSCs may attenuate secondary injury cascades, it exposes these cells to severe edema and inflammation, often leading to poor survival [10]. Furthermore, a key translational challenge with intravenous MSC therapy is the entrapment in the lung [11]. Therefore, along with NPC/NSCs, the subacute phase is also widely regarded as the optimal therapeutic window for MSC therapy [10].

Multilineage-differentiating stress-enduring (Muse) cells—stress-tolerant, non-tumorigenic endogenous macrophage-like/pluripotent-like stem cells—are expected to be a promising cell source for SCI treatment [12,13]. The term “pluripotent-like” refers to their capacity to differentiate into cells representative of all three germ layers, whereas “macrophage-like” refers to their ability to phagocytose damaged or apoptotic cellular fragments and use these signals to guide context-dependent differentiation [12,13]. Their accurate homing to the damaged site is mediated by the sphingosine-1-phosphate (S1P)–S1P receptor 2 (S1PR2) axis [14]. By sensing S1P, a universal tissue-damage signal, intravenously administered Muse cells can pass through the pulmonary capillaries and selectively migrate to the damaged tissues [14,15]. Upon reaching the lesion, they phagocytose damaged/apoptotic cellular fragments, and differentiate into the same cell type, thereby replacing damaged/dead cells and promoting structural and functional tissue repair [13,16,17,18,19,20,21]. This differentiation occurs spontaneously, guided by the differentiation factors directly recycled from engulfed injured/apoptotic cell fragments, without the need for external induction factors. Since Muse cells are pluripotent-like, they can differentiate into multiple cell types that comprise the tissue [22]. This mechanism of “physiologic reconstruction” fundamentally distinguishes them from MSCs, which differentiate only into osteogenic, chondrogenic, and adipogenic cells, deliver temporal bystander effect, and rarely engraft long-term [4,14]. Additionally, Muse cells exhibit robust immune privilege, characterized by the high human leukocyte antigen (HLA)-G expression and the absence of HLA-DR expression [14,18]. These features enable allogeneic administration without HLA matching or immunosuppression, a feasibility validated in multiple clinical trials, positioning Muse cells as a distinct and practical alternative [23,24,25,26,27,28].

While the phase 1 SCI clinical trial treated patients with intravenous Muse cell administration at 3 weeks post-injury, the optimal therapeutic window has not yet been verified [26]. This timing was selected based on the conventional consensus in the SCI field—which regards the subacute phase as optimal for conventional cell therapy—and results from Muse cell therapy for stroke studies, including clinical trials [15,24,29]. Specifically, a randomized, double-blind, placebo-controlled phase 2 trial evaluated the efficacy of intravenous Muse cells administered at the subacute phase (14–28 days post-stroke). The study demonstrated a significantly higher responder rate in the Muse cell group compared to the placebo group at 12 weeks, and statistically significant recovery in Fugl-Meyer motor score until 52 weeks. Based on these results, it was considered that intravenous Muse cell therapy retains therapeutic potential for central nervous system (CNS) injury even during this subacute time window [24]. However, simply applying this “subacute window” to Muse cell therapy for SCI may not be sufficient. This disparity likely reflects fundamental differences in the tissue architecture and intrinsic regenerative capacity between the brain and spinal cord [30]. The brain benefits from rich collateral circulation and intrinsic neurogenesis, providing a permissive environment for regeneration [31,32]; in contrast, the spinal cord is organized as a compact bundle of longitudinal tracts with limited collateralization and exhibits sustained inflammation, creating a hostile environment [30,33]. These biological differences suggest that, although both the brain and spinal cord are CNS, the optimal therapeutic window for SCI may differ from that of stroke [30]. Conversely, in myocardial infarction, Muse cells demonstrate robust repair when administered intravenously in the acute phase, coinciding with the peak of the S1P level in the plasma [14]. Notably, a critical distinction arises: unlike MSCs, which are often restricted to the subacute phase due to poor acute survival and pulmonary sequestration [11], Muse cells possess the stress tolerance and S1P-mediated homing mechanism to potentially target this early phase [14,34]. Therefore, elucidating Muse cell-specific therapeutic window in SCI—whether it aligns with the subacute paradigm of conventional cell therapy (NPC/NSCs and MSCs) for SCI and Muse cell therapy for stroke, or the acute paradigm of Muse cell therapy for myocardial infarction—is essential for successful clinical translation.

We aimed to achieve two primary objectives: first, to examine the therapeutic window for intravenous Muse cell administration in contusive SCI; and second, to compare therapeutic efficacy and lesion targeting among Muse cells, MSCs, and vehicle controls.

## 2. Results

### 2.1. Plasma S1P Dynamics

**Figure 1:**
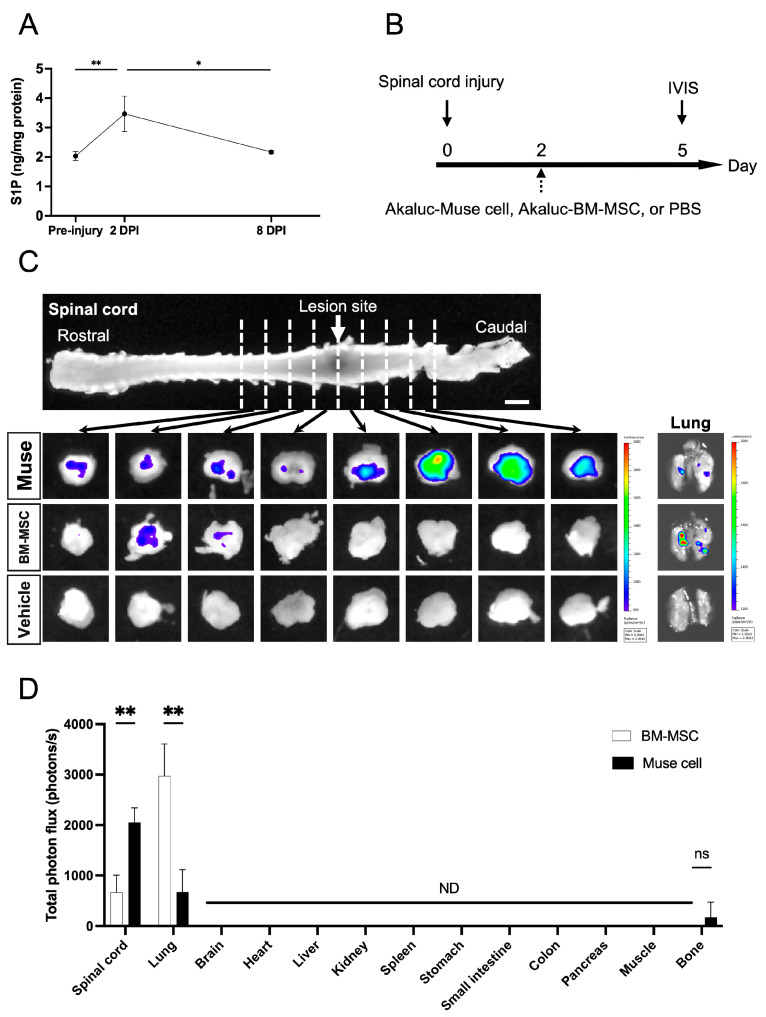
Plasma S1P dynamics and ex vivo bioluminescence imaging. (**A**) Plasma S1P concentrations (*n* = 3 per time point). S1P levels were maximum at 2-DPI. (**B**) Experimental protocol. Two days after spinal cord injury, the mice were intravenously administered Akaluc-Muse cells (5.0 × 10^4^ cells in 0.10 mL PBS), Akaluc-BM-MSCs (5.0 × 10^4^ cells in 0.10 mL PBS), or vehicle (PBS, 0.10 mL). Three days later, organs were removed and assessed using IVIS Spectrum computed tomography. (**C**) Representative ex vivo imaging of the spinal cord and lungs. (**D**) Quantification of photon flux in each organ (*n* = 3 per group). Signal was significantly higher in the spinal cord and significantly lower in the lungs of the Muse cell group compared with the BM-MSC group. Data are mean ± SEM. Symbols: * *p* < 0.05; ** *p* < 0.01; ns, not significant; ND, not detected. Scale bar in (**C**) =2 mm.

Because S1P is a key mediator of Muse cell homing after intravenous engraftment, we first examined temporal changes in plasma S1P levels after SCI (Figure 1A) [14]. Plasma S1P levels increased transiently, peaking at 2-DPI (3.5 ± 0.35 ng/mg protein) compared with pre-injury (2.0 ± 0.09 ng/mg protein, *p* = 0.007), and then declined toward baseline by 8-DPI (2.2 ± 0.03 ng/mg protein; 2-DPI vs. 8-DPI, *p* = 0.011).

### 2.2. In Vivo Dynamics of Injected Cells

Based on the 2-DPI peak in plasma S1P, we next compared the early biodistribution of intravenously injected human bone marrow-derived Muse cells and human bone marrow-derived mesenchymal stromal cells (BM-MSCs) using ex vivo bioluminescence imaging (Figure 1B). For this analysis, Akaluc-labeled Muse cells and BM-MSCs were used to track cell distribution. Ex vivo imaging showed that Akaluc-Muse cells were distributed along the injured spinal cord, with signal extending at least 8 mm rostral and caudal to the contusion epicenter 3 days after cell injection (Figure 1C). Photon flux at the perilesional site was higher in the Muse cell group than in the BM-MSC group (2051.1 ± 167.4 vs. 664.6 ± 198.4 photons/s, *p* = 0.006; Figure 1D). Lung signal was significantly lower in the Muse cell group than in the BM-MSC group (672.1 ± 254.4 vs. 2976.4 ± 364.5 photons/s, *p* = 0.007). A weak but detectable Muse cell signal was observed in the bone marrow, whereas no appreciable bioluminescence was detected in other organs (Figure S1). These data indicate that—in contrast to BM-MSCs, where the majority of intravenously injected cells were trapped in the lung—Muse cells bypass the pulmonary first-pass and preferentially home to the injured spinal cord.

### 2.3. Behavioral Recovery

**Figure 2:**
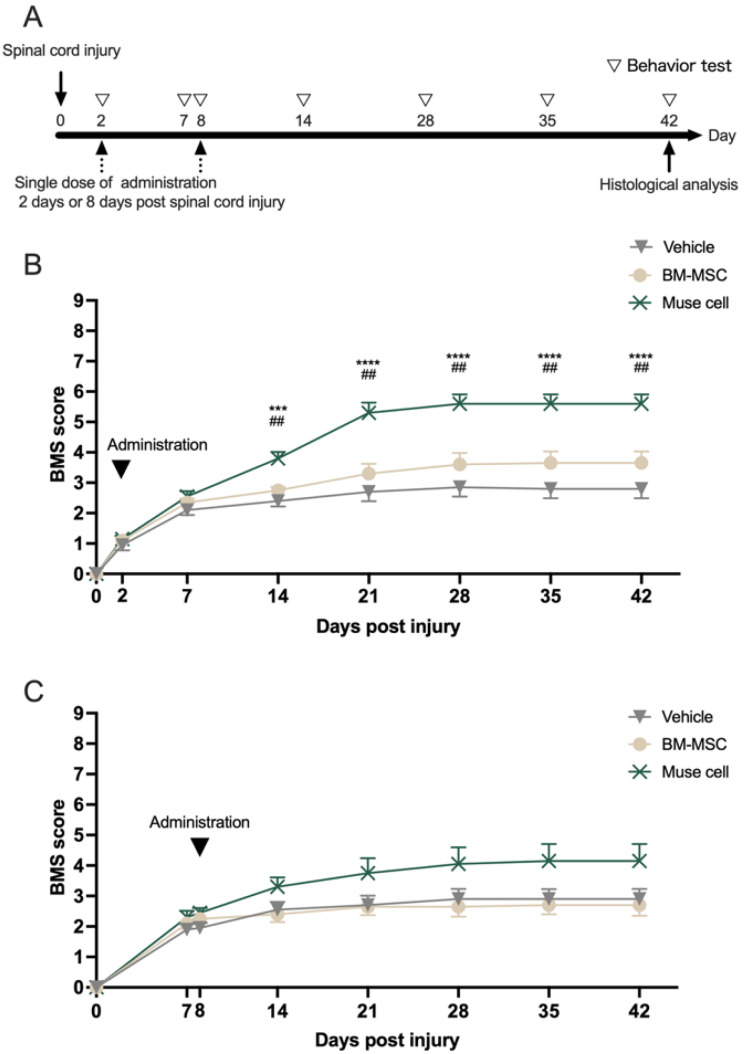
Behavioral outcomes in the 2- and 8-DPI treatment cohorts. (**A**) Experimental design. Mice with severe contusive spinal cord injury were allocated to cohorts receiving treatment at 2- or 8-DPI. Each cohort received a single tail-vein injection of human Muse cells (5 × 10^4^ cells in 0.10 mL PBS), human BM-MSCs (5 × 10^4^ cells in 0.10 mL PBS), or vehicle (0.10 mL PBS), and no immunosuppressants were used. Hindlimb locomotion was evaluated with the Basso Mouse Scale (BMS) on the administration day and weekly after injury. Tissue was collected at 42 days post-injury. (**B**) 2-DPI cohort (*n* = 10 per treatment group). The 2-DPI Muse cell group exhibited significantly higher BMS scores than the BM-MSC and vehicle groups at 14 days after injury, and this difference was maintained through 42 days. (**C**) 8-DPI cohort (*n* = 10 per treatment group). No significant differences were detected among the three groups during the observation period. Data are mean ± SEM. Symbols: Asterisks indicate Muse cell vs. vehicle comparisons: *** *p* < 0.001; **** *p* < 0.0001. Hash marks indicate Muse cell vs. BM-MSC comparisons: ## *p* < 0.01.

To determine whether the timing of cell administration affected locomotor recovery after SCI, we compared the Basso Mouse Scale (BMS) scores among the Muse cell, BM-MSC, and vehicle groups on the day of cell administration and weekly after SCI [35]. Time courses of BMS scores are shown in Figure 2A. Animals were divided into four treatment-timing cohorts: 2, 8, 14, and 28 DPI. Within each cohort, mice received Muse cells, BM-MSCs, or vehicle. Based on a previous classification of secondary injury responses in SCI, these time points correspond to the acute phase (2-DPI), subacute phase (8-DPI), transition between the subacute and chronic phases (14-DPI), and chronic phase (28-DPI), respectively [36,37]. In the 2-DPI cohort, two-way repeated-measures analysis of variance (ANOVA) with Geisser–Greenhouse correction showed significant effects of time (F(1.65, 44.49) = 208.20, *p* < 0.001), treatment group (F(2, 27) = 20.51, *p* < 0.001), and time × treatment group interaction (F(3.30, 44.49) = 12.66, *p* < 0.001). At the time of cell administration in the 2-DPI cohort, baseline BMS scores were comparable among the Muse cell, BM-MSC, and vehicle groups (1.2 ± 0.1, 1.1 ± 0.1, and 1.2 ± 0.1, respectively; Muse cell vs. vehicle, *p* > 0.999; Muse cell vs. BM-MSC, *p* > 0.999). In addition, actual impact parameters, including force, displacement, and velocity, did not differ among the treatment groups, indicating comparable initial injury severity (Figure S2). In the 2-DPI cohort, the Muse cell group showed progressive recovery, with BMS scores becoming significantly higher than those in the BM-MSC and vehicle groups at 14 days after injury and remaining significantly higher through 42 days. At 14 days after injury, the BMS score was 3.8 ± 0.2 in the Muse cell group, compared with 2.8 ± 0.1 in the BM-MSC group (*p* = 0.002) and 2.4 ± 0.2 in the vehicle group (*p* < 0.001). At 42 days after injury, the BMS score of the Muse cell group (5.6 ± 0.3) remained significantly higher than that of the BM-MSC group (3.7 ± 0.4, *p* = 0.003) and vehicle group (2.8 ± 0.3, *p* < 0.001) (Figure 2B). At this time point, the mean BMS difference was 1.9 points versus the BM-MSC group (95% confidence interval (CI): 0.7 to 3.2) and 2.8 points versus vehicle group (95% CI, 1.6 to 4.0). In the 8-DPI cohort, the Muse cell group showed modest recovery (4.2 ± 0.6), but this did not differ significantly from the BM-MSC (2.7 ± 0.4, *p* = 0.128) and vehicle groups (2.9 ± 0.3, *p* = 0.216) (Figure 2C).

In the 14- and 28-DPI cohorts, none of the treatment groups showed clear improvement in motor recovery through 42 days (Figure S3). Consequently, we focused on the 2- and 8-DPI cohorts in the following experiments.

### 2.4. Histologic Analysis

**Figure 3:**
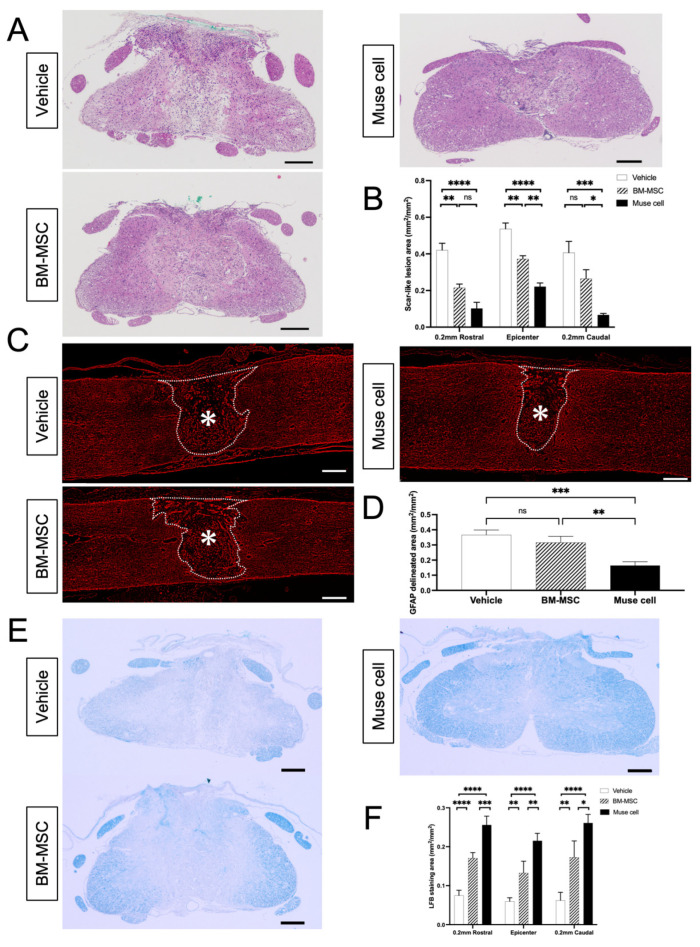
Histologic analysis (HE, GFAP, LFB) of spinal cord lesions in 2-DPI cohort at 42 days after spinal cord injury. (**A**) Representative HE staining at the lesion epicenter. (**B**) Scar-like lesion area quantification (*n* = 5 per treatment group). The scar-like lesion area was significantly reduced in the Muse cell group compared with the vehicle group at the epicenter and ± 0.2 mm, and compared with the BM-MSC group at the epicenter and 0.2 mm caudal, with a trend toward reduction at 0.2 mm rostral to the epicenter. (**C**) Representative GFAP-delineated area. The asterisk indicates the GFAP-delineated area. (**D**) Quantification of the glial scar area (*n* = 5 per treatment group). The glial scar area was significantly reduced in the Muse cell group compared with the BM-MSC and vehicle groups. (**E**) Representative LFB staining at the epicenter. (**F**) Quantification of spared myelin (*n* = 5 per treatment group). The significantly larger preserved myelin area was observed in the Muse cell group compared with the BM-MSC and vehicle groups. Data are mean ± SEM. Symbols: * *p* < 0.05; ** *p* < 0.01; *** *p* < 0.001; **** *p* < 0.0001; ns, not significant. Scale bars in (**A**,**C**,**E**) =200 µm.

To determine whether the functional improvement was accompanied by histological tissue preservation, we assessed lesion formation and myelin preservation at 42 days after SCI. Hematoxylin–eosin (HE) staining was used to quantify the scar-like lesion area (Figure 3A). In the 2-DPI cohort 42 days after SCI, the Muse cell group had smaller scar-like lesion areas at the epicenter and at 0.2 mm rostral and caudal to the epicenter compared with the vehicle group (epicenter, 0.2 mm rostral and caudal, *p* < 0.001) (Figure 3B). Compared with the BM-MSC group in the 2-DPI cohort, the scar-like lesion area of the Muse cell group was significantly smaller at the epicenter and 0.2 mm caudal (epicenter, *p* = 0.002; 0.2 mm caudal, *p* = 0.030), with a smaller trend observed at 0.2 mm rostral (*p* = 0.073). Evaluation of the 8-DPI cohort on day 42 revealed no significant differences in the scar-like lesion area among the three groups (Figure S4A,B).

To further assess lesion-associated astrogliosis, we evaluated the glial fibrillary acidic protein (GFAP)-defined glial scar area using immunohistochemistry (Figure 3C) [38]. At 42 days following SCI, in the 2-DPI cohort, the glial scar area of the Muse cell group was smaller than that in the BM-MSC and vehicle groups (Muse cell vs. BM-MSC, *p* = 0.007; Muse cell vs. vehicle, *p* < 0.001) (Figure 3D). In the 8-DPI cohort at 42 days after injury, no significant differences were observed in the glial scar area among the three groups (Figure S4C,D).

Luxol fast blue (LFB) staining was performed to assess myelin preservation (Figure 3E). At 42 days after injury, in the 2-DPI cohort, the Muse cell group had a larger spared-myelin area than the BM-MSC and vehicle groups at the epicenter and 0.2 mm rostral and caudal (Muse cell vs. BM-MSC: epicenter, 0.2 mm rostral and caudal, *p* < 0.001; Muse cell vs. vehicle: epicenter, 0.2 mm rostral and caudal, *p* < 0.001) (Figure 3F). In the 8-DPI cohort at 42 days after injury, no significant differences were observed in the spared-myelin area among the three groups (Figure S4E,F). These findings suggest that Muse cell treatment at 2-DPI was associated with reduced tissue damage and greater myelin preservation at 42 days after injury. In contrast, no significant differences in scar size or myelin preservation were observed among the three groups in the 8-DPI cohort (Figure S4).

### 2.5. Marker Expression in the Engrafted Cells

**Figure 4:**
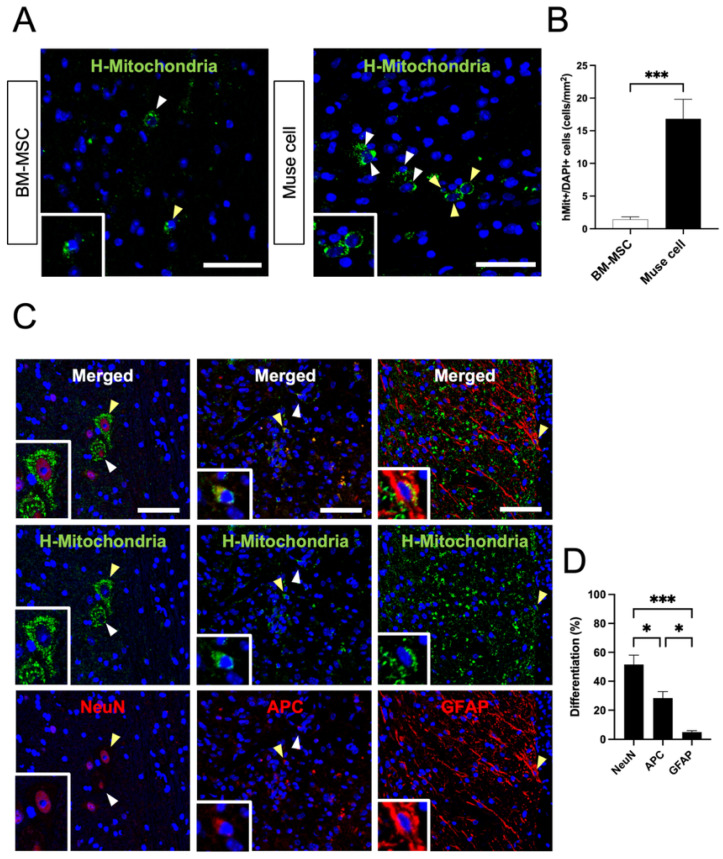
Muse cell engraftment and neural-lineage co-expression in the 2-DPI treatment cohort at 42 days after spinal cord injury. (**A**) Representative hMit/DAPI staining (white arrowheads) confirming human BM-MSC and human Muse cell engraftment; inset: high magnification (yellow arrowheads). (**B**) Quantification of hMit+/DAPI+ cells (*n* = 5 per treatment group). Counts were normalized to the analyzed spinal cord area. Muse cell group: 16.8 ± 3.0 cells/mm^2^; BM-MSC group: 1.4 ± 0.4 cells/mm^2^ (**C**) Representative neural-lineage differentiation into NeuN+, APC+, and GFAP+ cells (white arrowheads); inset: high magnification (yellow arrowheads). (**D**) Differentiation efficiencies of the Muse cell group (*n* = 5). NeuN 51.5 ± 6.5%, APC 28.4 ± 4.8%, GFAP 5.0 ± 1.0%. Data are mean ± SEM. Symbols: * *p* < 0.05; *** *p* < 0.001. Scale bars in (**A**,**C**) =50 µm.

To characterize the engraftment and phenotypic fate of transplanted human cells, we examined human mitochondria (hMit)-positive cells and their co-expression of neural-lineage markers at 42 days after SCI. The specificity of the anti-hMit antibody was first validated on human tissue sections as a positive control (Figure S5). To assess background signal, sections processed without the primary antibody were also included as negative controls, including human spinal cord tissue and spinal cord tissue from BM-MSC-treated and Muse cell-treated mice (Figure S5). These controls supported the use of hMit as a human-specific marker to identify human-derived Muse cells and BM-MSCs under the staining conditions used in this study [39]. At 42 days after injury, in the 2-DPI cohort, hMit+/4′,6-diamidino-2-phenylindole (DAPI)+ cells were detected around the contusion site in the Muse cell group (Figure 4A), suggesting Muse cell engraftment. The number of engrafted human cells was higher in the Muse cell group than in the BM-MSC group (16.8 ± 3.0 cells/mm^2^ vs. 1.4 ± 0.4 cells/mm^2^, *p* < 0.001) (Figure 4B). Double staining showed that hMit+/DAPI+ cells co-expressed neuronal nuclei (NeuN)+, adenomatous polyposis coli (APC)+, or GFAP+ cells, consistent with neural-lineage marker expression in vivo (Figure 4C). The proportions of each lineage were NeuN+ cells, 51.5 ± 6.5%; APC+ cells, 28.4 ± 4.8%; and GFAP+ cells, 5.0 ± 1.0% (Figure 4D). NeuN+ cells were the most abundant among the three lineages (NeuN+ cells vs. APC+ cells, *p* = 0.019; NeuN+ cells vs. GFAP+ cells, *p* < 0.001). The remaining hMit^+^/DAPI^+^ cells did not show detectable expression of NeuN, APC, or GFAP under the present staining conditions.

### 2.6. Serotonergic Input to the Lumbar Enlargement

**Figure 5:**
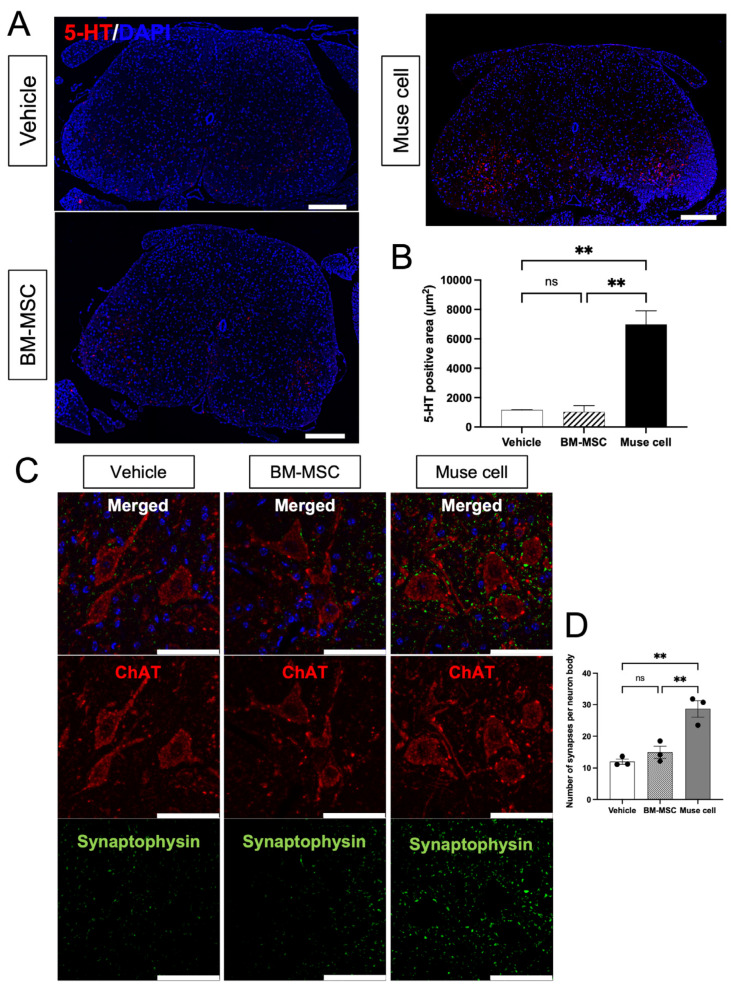
Lumbar enlargement in the 2-DPI cohort at 42 days after spinal cord injury: 5-HT-positive area and perisomatic synapses. (**A**) Representative immunofluorescence for 5-HT within the lumbar ventral horn. (**B**) Quantification of 5-HT-positive area (*n* = 3 per treatment group). Significantly larger 5-HT-positive areas were observed in the Muse cell group than in the BM-MSC and vehicle groups. (**C**) Representative perisomatic synapses apposed to ChAT-positive motor neurons. (**D**) Number of synapses per motor neuron (*n* = 3 per treatment group). One image per side (left/right) per animal was analyzed. More synapses were observed in the Muse cell group than in the BM-MSC and vehicle groups. Data are mean ± SEM. Symbols: ** *p* < 0.01. Scale bar in (**A**) =200 µm; scale bar in (**C**) =50 µm.

To examine whether Muse cell treatment was associated with preservation of descending serotonergic input, we quantified the 5-hydroxytryptamine (5-HT)-positive area in the ventral horn of the lumbar enlargement (Figure 5A). At 42 days after SCI, in the 2-DPI cohort, the 5-HT-positive area was significantly larger in the Muse cell group than in the BM-MSC and vehicle groups (Muse cell vs. BM-MSC: *p* = 0.001; Muse cell vs. vehicle: *p* = 0.001) (Figure 5B). In the 8-DPI cohort at day 42, no differences were detected among the three groups (Figure S6A,B).

### 2.7. Perisomatic Synapses in the Lumbar Enlargement

To further evaluate synaptic input to lumbar motor neurons, we quantified synaptophysin-positive puncta apposed to choline acetyltransferase (ChAT)-positive motor neurons in the lumbar enlargement. Confocal quantification showed an increase only in the 2-DPI cohort at day 42 (Figure 5C). In this cohort, the mean number of synaptic puncta per motor neuron was significantly higher in the Muse cell group than in the BM-MSC and vehicle groups (Muse cell vs. BM-MSC, *p* = 0.007; Muse cell vs. vehicle, *p* = 0.003) (Figure 5D). At 42 days following SCI, the 8-DPI cohort showed no significant difference among the three groups (Figure S6C,D).

### 2.8. Loss-of-Function Validation

**Figure 6:**
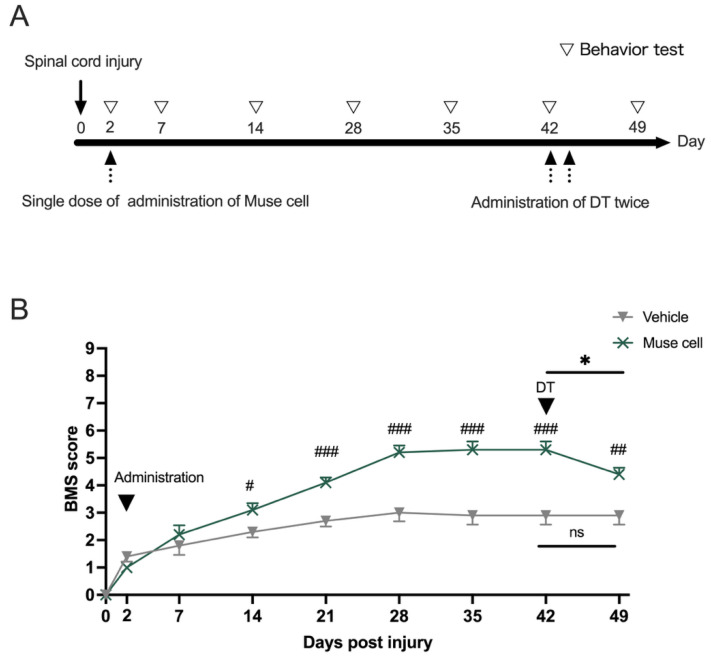
Experimental protocol and effects of DT ablation on locomotor recovery after Muse cell transplantation. (**A**) Experimental protocol. Mice were intravenously injected with Muse cells (5.0 × 10^4^ cells in 0.10 mL PBS) or vehicle (0.10 mL PBS) at 2-DPI. The BMS scores were assessed on the administration day and weekly after injury. At 42 days after injury, diphtheria toxin (DT; 50 µg/kg, intraperitoneally) was administered twice at a 24 h interval and behavior was reassessed on day 49. (**B**) Behavioral results (*n* = 5 per treatment group). The BMS scores were significantly higher in the Muse cell group than in the vehicle group on day 14, and this difference was maintained through 42 days. In the Muse cell group, BMS scores declined significantly after DT administration. Despite the administration of DT, the mean BMS scores of the Muse cell group remained significantly higher than those of the vehicle group at 49 days after injury. Data are mean ± SEM. Symbols: Asterisks indicate pre-DT vs. post-DT comparisons within the Muse cell group: * *p* < 0.05. Hash marks indicate Muse cell vs. vehicle comparisons: # *p* < 0.05, ## *p* < 0.01, and ### *p* < 0.001. ns, not significant.

To determine whether engrafted human Muse cells contributed to locomotor recovery in the 2-DPI cohort, we selectively ablated human-derived cells using diphtheria toxin (DT). At 42 days after SCI, mice treated with Muse cells or vehicle were given two intraperitoneal injections of DT 24 h apart (Figure 6A). Thus, the vehicle group served as a DT-treated non-engrafted control. At 49 days after SCI, even though BMS scores in the Muse cell group declined significantly relative to the pre-DT baseline (*p* = 0.030) (Figure 6B), the post-DT BMS scores remained significantly higher in the Muse cell group than in the vehicle group (*p* = 0.008). DT ablation partially reduced recovery, suggesting that engrafted Muse cells contributed to motor function, while the remaining benefit may reflect earlier paracrine or host-mediated effects.

## 3. Discussion

In the present study, we investigated the therapeutic window for intravenous Muse cell therapy in SCI across treatment-timing cohorts corresponding to the acute, subacute, subacute-to-chronic transition, and chronic phases. Intravenous delivery of Muse cells at 2-DPI led to significantly improved BMS scores compared to BM-MSCs and vehicle, whereas no significant differences were detected when treatment was initiated at 8-, 14-, or 28-DPI, suggesting that intravenous Muse cell therapy was most effective when administered during the acute post-injury phase under the present experimental conditions. At 2-DPI, plasma S1P peaked, and ex vivo imaging showed higher perilesional cord signal and reduced lung signal in the Muse cell group compared with the BM-MSC group, suggesting more efficient lesion-directed homing of Muse cells. Early Muse cell administration also reduced scar formation and preserved myelin, suggesting attenuation of secondary tissue damage. Engrafted cells positive for hMit+ also expressed NeuN+, APC+, and GFAP+, consistent with neural-lineage differentiation of Muse cells. In the lumbar enlargement, Muse cell treatment was associated with a larger 5-HT-positive area and a greater perisomatic synapse density. Finally, DT ablation at 42 days post-SCI partially reduced the functional recovery observed in the Muse cell group, while post-ablation BMS scores remained higher than those in the vehicle group. Together, these findings support a role for engrafted Muse cells in promoting motor function recovery and suggest that the acute post-injury phase represents the therapeutic window for Muse cell therapy in SCI. However, some mechanistic interpretations remain inferential because of several limitations, including the limited observation period, the use of a single cell dose, the absence of electrophysiological and sensory assessments, the lack of tract-tracing analysis, and the absence of serial cytokine and trophic-factor profiling.

### 3.1. Homing and Engraftment

In a mouse contusion SCI model, early intravenous administration of Muse cells (2-DPI) led to greater accumulation at the perilesional site and reduced pulmonary sequestration compared with BM-MSCs, indicating superior targeting to the injured cord; plasma S1P levels also peaked at 2-DPI. Previous studies have shown that Muse cells use the S1P–S1PR2 axis, whereas BM-MSCs rely on the SDF-1–CXCR4 pathway and undergo greater first-pass pulmonary sequestration [14,40]. Similar organ targeting has been reported in models of myocardial infarction, aortic dissection, and liver transplantation [14,17,18]. However, the present study did not directly test whether the S1P–S1PR2 axis causally regulates Muse cell homing after SCI. Previous studies in myocardial infarction models have experimentally demonstrated S1P–S1PR2-dependent homing of Muse cells. In a rabbit acute myocardial infarction model, both S1PR2 siRNA introduction into Muse cells and co-administration of Muse cells with an S1PR2 antagonist substantially reduced the homing of intravenously injected Muse cells to the post-infarct area [14]. In addition, S1PR2 agonist treatment enhanced Muse cell mobilization and promoted cardiac repair [41]. These findings support the biological plausibility of S1P–S1PR2-mediated Muse cell homing, but whether the same mechanism operates in the injured spinal cord remains to be determined. Future studies using S1PR2 inhibition, S1PR2-deficient or knockdown Muse cells, or S1PR2 agonist treatment in SCI models are needed. From a translational perspective, clinical trials of Muse cell-based therapy for SCI and other diseases have consistently adopted an intravenous dose of 15 million cells [23,24,25,26,27,28]. Notably, evidence from a randomized placebo-controlled trial in stroke patients suggested the therapeutic potential of Muse cells at this dose [24]. In contrast, MSC trials typically require approximately 100 million cells—nearly 10 times the dose used for Muse cells—supporting superior homing efficiency of Muse cells [42]. Despite the absence of immunosuppression, more engrafted human cells were present in the Muse cell group at 42 days in our study. While we did not quantify in vivo HLA-G or host immune-cell infiltration in the lesion, HLA-G-mediated immune privilege may contribute to Muse cell engraftment under xenogeneic conditions [14,43]. Nevertheless, the observed persistence of human Muse cells should be interpreted cautiously as a finding in a human-to-mouse xenograft model, and it does not directly establish the immune dynamics or engraftment magnitude expected in an allogeneic human clinical setting. Despite these limitations, robust homing permits effective delivery via non-invasive intravenous injection, differentiating Muse cells from NPC/NSC-based approaches that typically necessitate intralesional administration [44]. Within the lesion area, hMit+ cells differentiated predominantly into NeuN+ cells and APC+ cells when Muse cells were administered in the acute phase. In NPC/NSC-based approaches, the hostile inflammatory milieu of the acute phase typically restricts differentiation to glial rather than neuronal lineages [8,9]. The observed neuronal differentiation likely reflects the unique property of Muse cells, which determine their differentiation direction through the phagocytosis of damaged/apoptotic cellular fragments, allowing them to achieve context-appropriate differentiation even within the acute inflammatory milieu [16]. The remaining hMit+/DAPI+ cells did not express NeuN, APC, or GFAP under the present staining conditions and may include less differentiated cells or cells expressing other lineage markers, including vascular-lineage markers. Because such additional markers were not examined, their precise fate could not be determined in the present study.

### 3.2. Paracrine Mechanisms and Host Protection

The 2-DPI window coincides with the early secondary-injury phase—cytokine surge, neutrophil influx, and blood–spinal cord barrier disruption—when anti-inflammatory and barrier-restorative signals are most effective [3,5]. Muse cells may therefore protect the injured spinal cord through paracrine signaling during the acute phase. A previous study reported that Muse cells secrete significantly higher levels of paracrine mediators than MSCs [45]. Candidate factors include granulocyte-colony stimulating factor (G-CSF), interleukin-10, transforming growth factor-β, and hepatocyte growth factor [46,47,48,49]. Previous research demonstrated that G-CSF plays a central role in protecting neurons within the Muse cell system and has consistently shown neuroprotective and anti-inflammatory effects in preclinical models of SCI [46,47]. These actions are consistent with the reduced scar area and preserved myelin in the 2-DPI Muse cell group. These histologic findings were interpreted primarily as evidence of host tissue protection, rather than as direct evidence of de novo regeneration. Consistent with host tissue protection, DT ablation at 42 days post-injury partially reduced BMS recovery, and the post-ablation Muse cell group remained significantly above the vehicle group. This finding suggests that persistently engrafted Muse cells contributed, at least in part, to functional recovery, while the remaining benefit may reflect early paracrine actions during the acute secondary-injury phase and subsequent host tissue preservation. However, because cytokine and trophic factor profiling was not performed in the present study, the involvement of these specific paracrine factors remains inferential and should be interpreted with caution. At the lumbar enlargement, the expanded 5-HT-positive area and greater number of synaptophysin-positive puncta apposed to ChAT+ motor neurons likely reflect activity-dependent remodeling of spared host circuits. However, because tract-tracing was not performed, we cannot determine whether these changes reflect host-circuit remodeling, graft-related axonal relay, or other mechanisms. Conversely, in the 8-DPI cohort, the delayed administration likely missed this critical window for attenuating the secondary injury cascade, resulting in insufficient tissue repair and neuroprotection to improve behavioral outcomes. A prior study has reported beneficial effects of intravenous Muse cell therapy when administered in the subacute phase (e.g., 14 DPI); however, those findings were obtained under experimental conditions that differ from our study, including the use of immunosuppressive regimens, a different cell source (clinical-grade Muse cell product such as CL2020), a rat model, and injury paradigms that are not strictly comparable in severity [50,51]. Moreover, the 14-DPI time point was not directly compared with earlier administration within the same model, and it remains unclear from previous studies alone whether efficacy depends on the administration time point. In contrast, a key strength of the current study is the systematic within-model comparison of multiple administration time points under otherwise identical conditions, which enables a direct assessment of time-dependent efficacy and suggests that intravenous Muse cell therapy may be more likely to confer functional benefit when delivered in the acute phase than a later time points under the current design.

### 3.3. Different Therapeutic Time Windows of Muse Cell Therapy in the Spinal Cord and Brain

In our SCI model, therapeutic efficacy was confined to 2-DPI and was not detected at or beyond 8-DPI. A previous study in a mouse stroke model, however, reported significant recovery with Muse cell therapy even when administered 14 days after stroke [15]. This disparity may reflect fundamental differences in the tissue architecture and regenerative capacity between the brain and spinal cord. The brain’s rich vascular perfusion and collateral circulation help sustain the peri-infarct penumbra [31], while its complex redundant neural networks and intrinsic neurogenesis provide a more permissive environment for regeneration [32]. In contrast, the spinal cord has limited collateralization and is organized as a compact bundle of longitudinal tracts [32]. It also exhibits sustained inflammation and early glial scarring, both of which create a hostile environment for regeneration [33]. In stroke, a favorable environment may facilitate Muse cell-mediated regeneration beyond the subacute phase [39]. Importantly, the randomized clinical trial for stroke confirmed that the subacute brain microenvironment remains receptive to repair, as evidenced by a significantly higher responder rate in the Muse cell group compared to the placebo group [24]. While this study provided clinical proof that Muse cells retain therapeutic potential in the subacute phase of stroke, our findings indicate that this “subacute window” does not translate to the spinal cord. Although the Phase 1 SCI trial for Muse cell therapy successfully met its primary endpoint of safety and demonstrated feasibility, when compared with our present findings, the injection window in the phase 1 trial may not have coincided with the optimal period for maximizing therapeutic potential [26]. For phase 2/3 trials performed primarily to evaluate efficacy, earlier injection within the acute phase should be considered.

### 3.4. Clinical Implications

Our findings support intravenously administered Muse cells as a practical candidate for acute SCI therapy. Even in the acute phase, the cells selectively homed to the lesion site, unlike MSCs that were known to largely be entrapped in the lung after intravenous injection [11]. In addition, the ability to administer allogeneic Muse cells intravenously without immunosuppression offers a broadly accessible option that could be implemented even in hospitals without on-site spine surgery services, unlike NPC/NSC-based strategies that rely on specialized surgical delivery [43,52]. Clinical studies of olfactory mucosa autograft transplantation have also reported neurological improvement in some patients with chronic SCI; however, this approach requires surgical transplantation of autologous tissue into the lesion site and therefore differs substantially from the intravenous Muse cell strategy evaluated in the present study [53,54,55].

For future clinical translation, it is notable that the earlier administration within the acute–early subacute window may be important. Although rodent DPI cannot be directly translated to human clinical timelines, comparative analyses of inflammatory responses indicate that 2-DPI in rodents roughly corresponds to 5–7 days post-injury in humans [56]. However, this rodent-to-human timing comparison should be regarded as hypothesis-generating rather than as a direct clinical recommendation, because injury time course, injury severity, acute medical instability, and the feasibility of cell-product preparation and delivery differ substantially between rodents and humans. In this context, administration earlier than the timing used in the first-in-human trial (approximately 3 weeks post-injury) may merit evaluation.

### 3.5. Limitations

First, the observation period may have been insufficient to fully evaluate long-distance neural regeneration. The primary objective of this study, however, was to define the therapeutic window for Muse cell therapy rather than to capture the entirety of long-term circuit reconstruction. Because locomotor recovery had already stabilized and histologic outcomes were consistent with the behavioral findings, the observation period was considered adequate to detect treatment effects. Nevertheless, longer-term observations are necessary to further elucidate the therapeutic mechanisms and neuronal circuit reconstruction of Muse cells in SCI. We evaluated only a single intravenous dose (5 × 10^4^ cells) given as a single infusion; repeat-dosing effects were not assessed. The 14- and 28-DPI cohorts included fewer animals than the 2- and 8-DPI cohorts and were therefore underpowered. Thus, the negative findings in these later time-window cohorts should be interpreted cautiously. In addition, no formal interim-analysis plan or pre-specified stopping criteria were defined for the exploratory 14- and 28-DPI cohorts; therefore, their early termination should be interpreted as a pragmatic decision rather than a formal futility analysis. Moreover, because this study did not include a dedicated chronic SCI model or quantify putative late-phase determinants of treatment efficacy, the present findings cannot define the therapeutic potential of Muse cell therapy in chronic SCI. Only female C57BL/6J mice were used; therefore, whether the present findings apply to male mice remains to be determined. Functional outcomes centered on BMS locomotion, and electrophysiologic and sensory endpoints were not collected, limiting the resolution of circuit and sensory recovery. Additionally, several mechanistic endpoints, including S1P measurement, IVIS imaging, histologic quantification, 5-HT-positive area, perisomatic synapse density, and DT ablation analysis, were exploratory and were not independently powered; therefore, these findings should be interpreted with caution. Tract-tracing and ultrastructural analyses were not performed; therefore, the present study does not provide direct evidence for graft-derived axonal relay, remyelination, or true axonal regeneration. Definitive confirmation of neural-lineage differentiation of engrafted human cells requires orthogonal validation using approaches such as human nuclear antigen staining, human-specific in situ hybridization, genetic reporters, or confocal z-stack analysis. Serial in vivo profiling of cytokines and trophic factors was not performed; thus, the proposed paracrine attributions remain inferential. Although plasma S1P levels peaked at 2-DPI and Muse cells showed enhanced homing to the injured spinal cord at this time point, the present study did not directly test the causal role of the S1P–S1PR2 axis. Future studies using S1PR2 inhibition, S1P neutralization, or S1PR2-deficient/knockdown Muse cells are required to determine whether this pathway is necessary for Muse cell homing and functional recovery after SCI. Because human cells were transplanted into immunocompetent mice without immunosuppression, the immune dynamics of this human-to-mouse xenograft model may not fully recapitulate those of allogeneic Muse cell administration in humans. We also did not quantify host immune-cell infiltration, such as CD3+ T cells or CD68+ macrophages, at the lesion site. Our comparative analysis demonstrated that Muse cells exhibit superior acute-phase homing compared with MSCs and, distinct from NPC/NSCs, differentiate into neural lineage cells via a phagocytosis-mediated mechanism even when administered in the acute phase [16]. These findings offer foundational data to guide the temporal design of future clinical trials. Furthermore, this study supports the concept that the optimal therapeutic window may depend on the biological properties of the specific cell type, although the underlying mechanisms require further validation.

## 4. Materials and Methods

### 4.1. Animal

All procedures were approved by the Institutional Animal Care and Research Advisory Committee of our institute and are reported in accordance with the ARRIVE 2.0 guidelines.

Female C57BL/6J mice (8–9 weeks old, 20–25 g; CLEA Japan, Tokyo, Japan) were used. Mice were housed under standard conditions (22–24 °C; 12 h light/dark cycle) with ad libitum access to food and water, and animal welfare was monitored daily with refinements applied as needed to minimize pain and distress. Female C57BL/6J mice were used to reduce sex-related variability and to facilitate postoperative management after SCI.

### 4.2. Spinal Cord Injury Model

The mice were anesthetized by intraperitoneal injection of a combination of three anesthetic agents: medetomidine (0.30 mg/kg), midazolam (4.0 mg/kg), and butorphanol (5.0 mg/kg) [57]. After a midline dorsal incision, a T9 laminectomy was performed, and a severe contusive injury (70 kdyn) was delivered using an Infinite Horizon impactor (IH impactor; Precision Systems & Instrumentation, Lexington, KY, USA) [58]. Impact parameters and force delivery were verified by the device software. Cefazolin (15 mg/kg) was administered subcutaneously once daily for 7 days [58]. Bladders were expressed manually twice daily until spontaneous voiding returned. Animals were humanely killed at predefined endpoints. At experimental endpoints, mice were fixed by transcardial perfusion (left ventricle) under deep anesthesia for tissue collection. In case of severe neuropathic pain (e.g., alopecia and autotomy/self-injury) or more than 20% loss of body weight from the pre-injury baseline, animals were euthanized by CO_2_ inhalation. The investigator who induced SCI was not involved in behavioral scoring, histologic quantification, or IVIS imaging analysis.

### 4.3. Preparation of Human Muse Cells and BM-MSCs

Human BM-MSCs employed in this study were obtained from Lonza Inc. (Allendale, NJ, USA). As stated in the supplier’s documentation, human source tissues were acquired under Institutional Review Board-approved protocols and with written informed consent from donors [59]. Human BM-MSCs were expanded under standard culture conditions. SSEA-3+ Muse cells were isolated by fluorescence-activated cell sorting (FACS) as previously described [12,60]. For the main therapeutic experiments, unlabeled human Muse cells and human BM-MSCs were used. Cells were washed and resuspended at 5 × 10^4^ cells in 0.10 mL phosphate-buffered saline (PBS) for tail vein injection. Because the same investigator prepared the cell suspensions and performed intravenous dosing, blinding during cell preparation and treatment administration was not fully implemented.

### 4.4. Experimental Design and Group Allocation

The experimental unit was a single mouse for all behavioral, imaging, and histological analyses. A total of 95 female C57BL/6J mice were allocated to four single-dose cohorts: 2-, 8-, 14-, or 28-DPI groups. These administration time points were selected to cover the major post-injury phases after SCI. Previous reviews have classified rodent SCI phases with slightly different temporal boundaries, depending on whether inflammatory responses, neural tissue degeneration, or glial scar maturation are emphasized [36,37]. Based on these classifications, the 2-, 8-, 14-, and 28-DPI cohorts were interpreted as corresponding to the acute phase, subacute phase, transition between the subacute and chronic phases, and chronic phase, respectively. Thus, this design allowed us to evaluate Muse cell administration across post-injury phases from acute to chronic SCI. Within each cohort, animals were assigned to receive human Muse cells (5 × 10^4^ cells suspended in 0.10 mL PBS), human BM-MSCs (5 × 10^4^ cells in 0.10 mL PBS), or vehicle (0.10 mL PBS) via tail-vein injection in accordance with previous reports [15], and no immunosuppressive agents were used. Group allocation within each DPI cohort was performed using a computer-generated block randomization list to balance the number of animals among the Muse cell, BM-MSC, and vehicle groups. The 14- and 28-DPI cohorts were exploratory later time-window cohorts. From the outset, behavioral recovery was reviewed after the first five animals per group had completed assessment; however, no formal statistical interim-analysis plan or pre-specified stopping criteria were defined. Because no clear treatment-related recovery trend was observed, no additional animals were added, and the final sample size for these cohorts was five animals per treatment group. Of the 95 mice enrolled, 90 completed behavioral assessment and were included in the primary analysis. Five mice were omitted from the main analysis because of death (the 2-DPI BM-MSC group, *n* = 1; the 8-DPI vehicle group, *n* = 1; the 28-DPI Muse cell group before treatment, *n* = 1), humane euthanasia due to excessive body-weight loss (the 8-DPI BM-MSC group, *n* = 1), or unsuccessful cell administration (the 2-DPI Muse cell group, *n* = 1). The final behavioral analysis included 10 mice per treatment group in the 2- and 8-DPI cohorts and 5 mice per treatment group in the 14- and 28-DPI cohorts. Behavioral assessments and histologic quantifications were performed by investigators blinded to the treatment group.

### 4.5. Behavioral Analysis

Hindlimb motor function was evaluated using the BMS scores for locomotion on the day of cell administration and weekly thereafter following SCI [35]. Each mouse was observed individually for 4 min in an open field by two blinded investigators. Hindlimb locomotor function was scored on the BMS scale from 0–9. BMS scores for the left and right hindlimbs were averaged to obtain a single value per test for each mouse at each time point. Outcome assessments were conducted by investigators blinded to group allocation.

### 4.6. Plasma S1P Quantification

Whole blood was collected via cardiac puncture from mice anesthetized by intraperitoneal injection of a combination of medetomidine, midazolam, and butorphanol as described above, at pre-injury, 2-DPI, and 8-DPI. Blood was anticoagulated with ethylenediaminetetraacetic acid and centrifuged at 4 °C to obtain plasma. Aliquots were deproteinized with ice-cold methanol containing 0.1 µM S1P(d17:1) as an internal standard, and S1P was quantified by reverse-phase high-performance liquid chromatography using an L-column 3 ODS column (3 µm, 2.0 × 50 mm; Chemicals Evaluation and Research Institute, Tokyo, Japan) coupled to a QTRAP 5500 mass spectrometer (Sciex Inc., Framingham, MA, USA). The column temperature was 40 °C and the flow rate was 0.2 mL/min. The mobile phases consisted of solvent A (acetonitrile:methanol:water, 1:1:3, *v*/*v*/*v*, containing 5 mM ammonium acetate) and solvent B (isopropanol containing 5 mM ammonium acetate). The gradient protocol was as follows: 0–1 min, 95% A; 1–9 min, linear increase of B from 5–95%; 9–13 min, 95% B. S1P(d18:1) was detected in positive ion mode with m/z transitions at 380/264 and quantified using MultiQuant version 2.0 (Sciex). Total plasma protein was measured by bicinchoninic-acid assay, and S1P values were normalized to protein content and expressed as ng/mg protein.

### 4.7. Ex Vivo Bioluminescence Imaging to Confirm Muse Cell Homing

Akaluc/pcDNA3, provided by Dr. S. Iwano (Laboratory for Cell Function and Dynamics, Brain Science Institute, RIKEN, Saitama, Japan), was subcloned into pWPXL to create pWPXL-Venus-Akaluc [61]. pWPXL-Venus-Akaluc, pMD2.G, and pCMVΔR8.74 were co-transfected into LentiX-293T packaging cells (Takara Bio, Shiga, Japan) with Lipofectamine 2000 (Thermo Fisher Scientific, Tokyo, Japan). After 72 h, the viral supernatant was collected, centrifuged, and passed through a 0.45 μm filter. Human BM-MSCs were infected with the supernatant [62]. Venus+ cells were isolated by fluorescence-activated cell sorting and designated as Akaluc-BM-MSCs. These cells were stained with rat anti-SSEA-3 IgM (1:1000, BioLegend, San Diego, CA, USA) and APC-conjugated goat anti-rat IgM (1:100, Jackson ImmunoResearch Laboratories, West Grove, PA, USA). Venus+/SSEA-3+ double-positive cells were collected as Akaluc-Muse cells and resuspended at 5 × 10^4^ cells in 0.10 mL PBS for tail vein injection. Two days after SCI, Akaluc-Muse cells, Akaluc-BM-MSCs, or vehicle were administered (Figure 1B). Three days after the cell injection, the mice were administered 1 mg of AkaLumine-HCl (FUJIFILM Wako Pure Chemical Corporation, Osaka, Japan) in normal saline via the tail vein [63]. Five minutes later, the mice were killed by administration of a lethal dose of isoflurane anesthesia, and each organ was dissected out. The entire spinal cord was sectioned into consecutive 2 mm axial slices. Each organ was immersed in 500 µM AkaLumine-HCl in normal saline and evaluated using an IVIS Spectrum computed tomography in vivo imaging system (IVIS; PerkinElmer, Waltham, MA, USA). Total photon flux (photons/s) was quantified with Living Image v4.7 (PerkinElmer) to assess cell homing. IVIS imaging analysis was performed using coded images by an investigator blinded to treatment allocation.

### 4.8. Histologic Analysis by HE and LFB Staining

At 42 days after SCI, mice in the 2- and 8-DPI cohorts were anesthetized as described above and transcardially perfused with cold saline followed by 4% paraformaldehyde. Spinal cords were removed, postfixed overnight in 4% paraformaldehyde, dehydrated, cleared, and embedded in paraffin using standard procedures. Paraffin sections (4 µm) were cut in the sagittal or axial plane. HE and LFB staining were used to evaluate gross morphology [64]. Digital images were acquired with a bright-field microscope (BZ-X710; Keyence, Osaka, Japan). Scar-like lesion and spared myelinated white-matter areas were evaluated in ImageJ software version 1.54i (National Institutes of Health, Bethesda, MD, USA) and normalized to the total area of the examined spinal cord section. Serial sections were analyzed at 0.2 mm intervals rostral and caudal to the lesion epicenter (defined as 0 mm). Five mice per treatment group were assessed in the 2- and 8-DPI cohorts. Quantification of the scar-like lesion area and spared myelin area was performed using coded tissue sections by an investigator blinded to treatment allocation.

### 4.9. Immunohistochemistry

Muse cell engraftment and lineage identification were evaluated by deparaffinizing paraffin sections in xylene, rehydrating them through graded ethanols, and blocking for 30 min with 20% Blockace, 5% bovine serum albumin, and 0.3% Triton X-100 in PBS at room temperature (RT). The sections were incubated overnight at 4 °C with anti-mitochondrial ribosomal protein L11 (hMit, a marker for human mitochondria; 1:500, #ab133789, Abcam, Cambridge, UK), followed by a 2 h incubation at RT with Alexa Fluor 488-conjugated donkey anti-rabbit IgG (1:200, #711-545-152; Jackson ImmunoResearch, West Grove, PA, USA). The same sections were then incubated overnight at 4 °C with mouse anti-NeuN (1:100, #ab177487; Abcam), mouse anti-GFAP (1:200, #G3893, Sigma-Aldrich, St. Louis, MO, USA), or mouse anti-APC (1:250, #ab16794, Abcam). The following day, the sections were incubated with Alexa Fluor 594-conjugated donkey anti-mouse IgG (1:200, #715-585-150; Jackson ImmunoResearch) for 2 h at RT. Nuclei were counterstained with 4′,6-diamidino-2-phenylindole (DAPI; 1:500, #D1306; ThermoFisher Scientific, Waltham, MA, USA). Images were captured on a Nikon A1 confocal microscope (Nikon Corporation, Tokyo, Japan). Counts of hMit+/DAPI+ cells were normalized to the spinal cord area and reported as cells/mm^2^.

Multiplex tyramide-signal-amplified immunofluorescence was performed using the Opal 7-Color Manual IHC kit (Akoya Biosciences, Inc., Hopkinton, MA, USA) to evaluate the lumbar enlargement circuitry [65]. The slides were baked at 65 °C for 1 h, then dewaxed and dehydrated. Endogenous peroxidase activity was quenched with 3% H_2_O_2_ during a 15 min incubation at RT. Antigen retrieval was performed in AR6 or AR9 buffer at 95–100 °C for 15 min, followed by blocking with Blocking One Histo (Nacalai Tesque, Kyoto, Japan) for a 10 min incubation at RT. Primary antibodies were applied overnight at 4 °C in antibody diluent: anti-GFAP (1:200, #G3893, Sigma-Aldrich), anti-5-hydroxytryptamine (5-HT) (a serotonergic fiber marker, 1:10,000, # 20080, Immunostar, Hudson, WI, USA), anti-choline acetyltransferase (ChAT, a motor neuron marker; 1:500, #ab178850, Abcam), and anti-synaptophysin (a presynaptic terminal marker; 1:500, #ab32127, Abcam). After washing in Tris-Buffered Saline with Tween 20, the sections were treated with anti-mouse/rabbit Opal Polymer HRP for 30 min at RT, and fluorophores were deposited by tyramide signal amplification: Opal 520 for GFAP, ChAT, and 5-HT, and Opal 620 for synaptophysin. Staining cycles were repeated, with heat-mediated antigen retrieval performed between rounds to strip bound antibodies. Slides were counterstained with DAPI and mounted. Multiplex images were acquired on a Leica THUNDER wide-field fluorescence system (Leica Microsystems CMS GmbH, Wetzlar, Germany), and ImageJ was used to measure (i) the GFAP-delineated lesion area, (ii) the 5-HT-positive fiber area in the lumbar ventral horn, and (iii) the number of synapses contacting ChAT-positive motor neurons. Immunohistochemical quantification was performed using coded tissue sections by an investigator blinded to treatment allocation.

### 4.10. Quantification of Glial Scarring Delineated by GFAP Immunostaining

Reactive astrocytes form a GFAP-positive rim enclosing the inflammatory core (GFAP-delineated lesion area corresponding to the glial scar) [38]. On mid-sagittal sections, the rim was traced using ImageJ and normalized to the total spinal cord cross-sectional area within ±0.5 mm of the lesion epicenter. For each animal, three sections closest to the epicenter were analyzed, and the values were averaged. Measurements were obtained for each treatment cohort (*n* = 5 per treatment group).

### 4.11. Quantification of Preserved 5-HT Fibers

The 5-HT-positive area within the ventral horn of the lumbar enlargement was quantified under 20× magnification, and positive areas on axial sections were measured using ImageJ [66]. Each section was imaged as a 6-panel (2 × 3) mosaic using LAS X Navigator (Leica Microsystems CMS GmbH, Wetzlar, Germany); tiles were stitched in LAS X to generate a seamless composite image, which was exported for quantification. In the 2- and 8-DPI cohorts, two sections were randomly selected from each of three animals per treatment group for analysis. The two section-level measurements were averaged within each animal, and the animal-level mean was used as a single data point for statistical analysis.

### 4.12. Quantification of Perisomatic Synaptic Contacts in the Lumbar Enlargement

Axial sections of the lumbar enlargement were imaged using a Leica THUNDER microscope (Leica Microsystems CMS GmbH, Wetzlar, Germany) equipped with a 100×/1.40 NA oil objective. Z-stacks were acquired at 1-µm intervals. The left and right ventral horn fields containing ChAT-positive motor neurons were analyzed in each mouse. Synaptophysin-positive puncta adjacent to each soma or proximal dendrite were counted manually [67]. Values from the left and right ventral horn fields were averaged within each animal, and the animal-level mean number of synapses per neuronal soma was used as a single data point for statistical analysis in the 2- and 8-DPI cohorts (*n* = 3 per treatment group).

### 4.13. Loss-of-Function Study

In the 2-DPI cohort, xenografted human cells were selectively ablated with DT (Sigma-Aldrich, St Louis, MO, USA) as described previously [68]. Compared with rodent cells, human cells are ~10^5^-fold more sensitive to DT; therefore, the toxin can be used to selectively eliminate engrafted human-derived cells [69,70]. Two days after spinal cord injury, either Muse cells (5 × 10^4^ cells in 0.10 mL PBS; *n* = 6) or vehicle control (0.10 mL PBS; *n* = 5) was administered via the tail vein. One mouse in the Muse cell group died during the experimental period; therefore, the final analysis included 5 Muse cell-treated mice and 5 vehicle-treated mice. Six weeks after SCI, the Muse cell and vehicle control groups received two intraperitoneal injections of DT (50 µg/kg) 24 h apart. Behavioral testing was performed 49 days after SCI (Figure 6A).

### 4.14. Statistical Analysis

All statistical analyses were performed in GraphPad Prism 10.0 (GraphPad Software, San Diego, CA, USA). Sample size was estimated a priori from historical behavioral data using BMS score at 6 weeks post-injury as the primary outcome, with power set at 0.80 and α = 0.05, yielding 10 animals per group. Continuous variables are expressed as the mean ± standard error of the mean (SEM). For repeated BMS measurements, two-way repeated-measures ANOVA was performed without assuming sphericity, with Geisser–Greenhouse correction, followed by Bonferroni’s multiple comparisons test. For endpoints with small sample sizes, including the 5-HT-positive area and the number of synapses in the lumbar enlargement, group differences were assessed using the Kruskal–Wallis test followed by Dunn’s multiple comparisons test. For all remaining endpoints—plasma S1P concentrations, scar-like tissue, spared myelinated area, GFAP-delineated area, and neural-lineage differentiation—group differences were assessed by one-way ANOVA, with Bonferroni post hoc testing applied when comparing three groups. Two-group comparisons, such as photon flux in IVIS imaging, were analyzed using the *t*-test. A two-tailed *p*-value below 0.05 was considered statistically significant. Exact analyzed sample sizes are indicated in the figures and legends. Statistical analysis was performed after completion of all outcome measurements; however, the statistical analysis itself was not performed under blinded conditions.

## 5. Conclusions

This study suggests that the early post-injury phase is a favorable therapeutic window for intravenous Muse cell therapy following contusive SCI in mice. Intravenous Muse cell therapy delivered functional and histologic benefits when administered at 2-DPI, while administration at 8-, 14-, and 28-DPI did not result in significant functional or histologic improvements compared with the BM-MSC and vehicle groups. Plasma S1P levels peaked at 2-DPI, and ex vivo imaging showed superior homing of Muse cells to the injured spinal cord, with a higher perilesional signal and lower pulmonary sequestration compared with BM-MSCs. Furthermore, early Muse cell administration mitigated secondary injury—evidenced by reduced scarring and preserved myelin—and achieved robust engraftment with neural-lineage differentiation, a functional contribution supported by DT ablation analysis. These results provide critical preclinical evidence to inform therapeutic windows and delivery timing in future clinical trials, supporting the evaluation of earlier administration schedules. These findings provide preclinical evidence supporting further investigation of acute-phase intravenous Muse cell therapy for SCI. They also highlight the unique biological characteristics of Muse cells compared with conventional stem cells and suggest that therapeutic timing should be optimized according to cell type-specific properties.

## Data Availability

The original contributions presented in this study are included in the article/Supplementary Material. Further inquiries can be directed to the corresponding authors.

## Supplementary Materials

The following supporting information can be downloaded at: https://www.mdpi.com/article/10.3390/ijms27146219/s1.

## Abbreviations

The following abbreviations are used in this manuscript:

APC	adenomatous polyposis coli
BMS	Basso Mouse Scale
BM-MSCs	bone marrow-derived mesenchymal stromal cells
ChAT	choline acetyltransferase
CNS	central nervous system
DAPI	4′,6-diamidino-2-phenylindole
DT	diphtheria toxin
DPI	days post-injury
GFAP	glial fibrillary acidic protein
HE	hematoxylin–eosin
5-HT	5-hydroxytryptamine
HLA	human leukocyte antigen
hMit	human mitochondria
LFB	luxol fast blue
Muse cells	multilineage-differentiating stress-enduring cells
NeuN	neuronal nuclear protein
NPC	neural progenitor cell
NSC	neural stem cell
MSCs	mesenchymal stromal cells
PBS	phosphate-buffered saline
RT	room temperature
SCI	spinal cord injury
SEM	standard error of the mean
S1P	sphingosine-1-phosphate
S1P–S1PR2 axis	sphingosine-1-phosphate–sphingosine-1-phosphate receptor-2 axis
