# Supplementary material for: Therapeutic Window for Intravenous Human Muse Cell Administration in Mouse Spinal Cord Injury
Source: Int J Mol Sci. 2026 Jul 12;27(14):6219. doi: 10.3390/ijms27146219 (PMC13409780; doi:10.3390/ijms27146219)
Supplement: Supplementary file 1 [file ijms-27-06219-s001.zip › ijms-4367326-supplementary.pdf]

Supplementary Materials

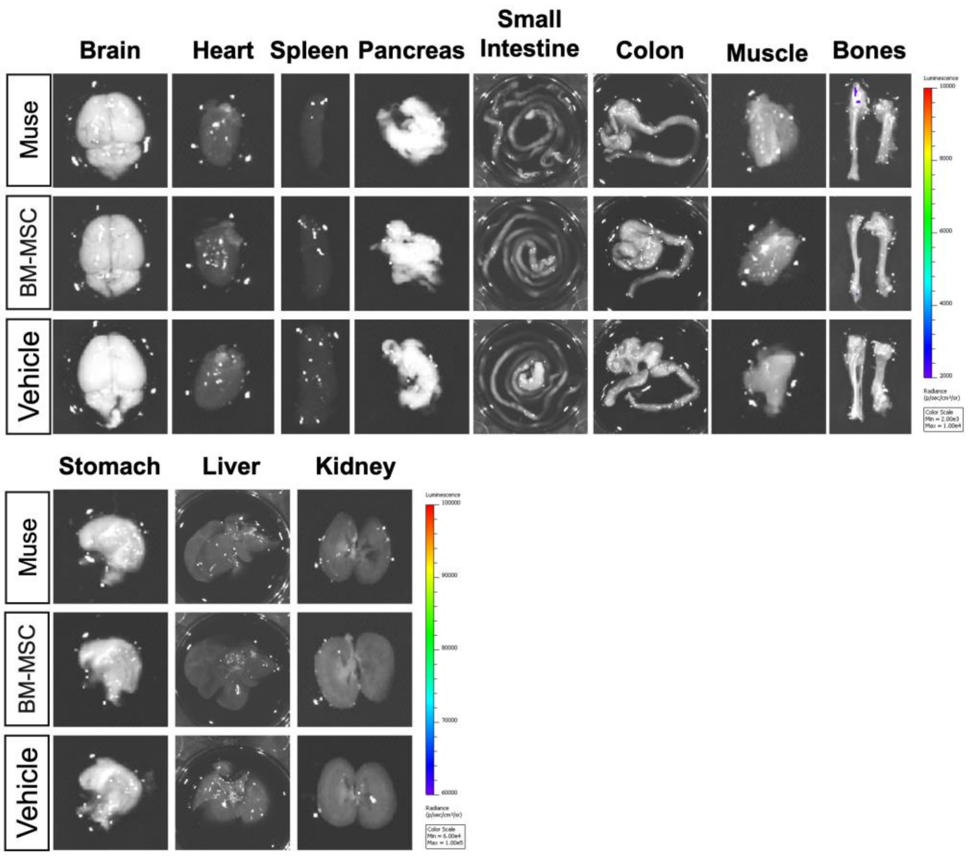

Figure S1 Ex vivo bioluminescence in non-spinal organs

A weak but detectable Akaluc-Muse cell signal was observed in bone marrow; no bioluminescence was detected in other organs.

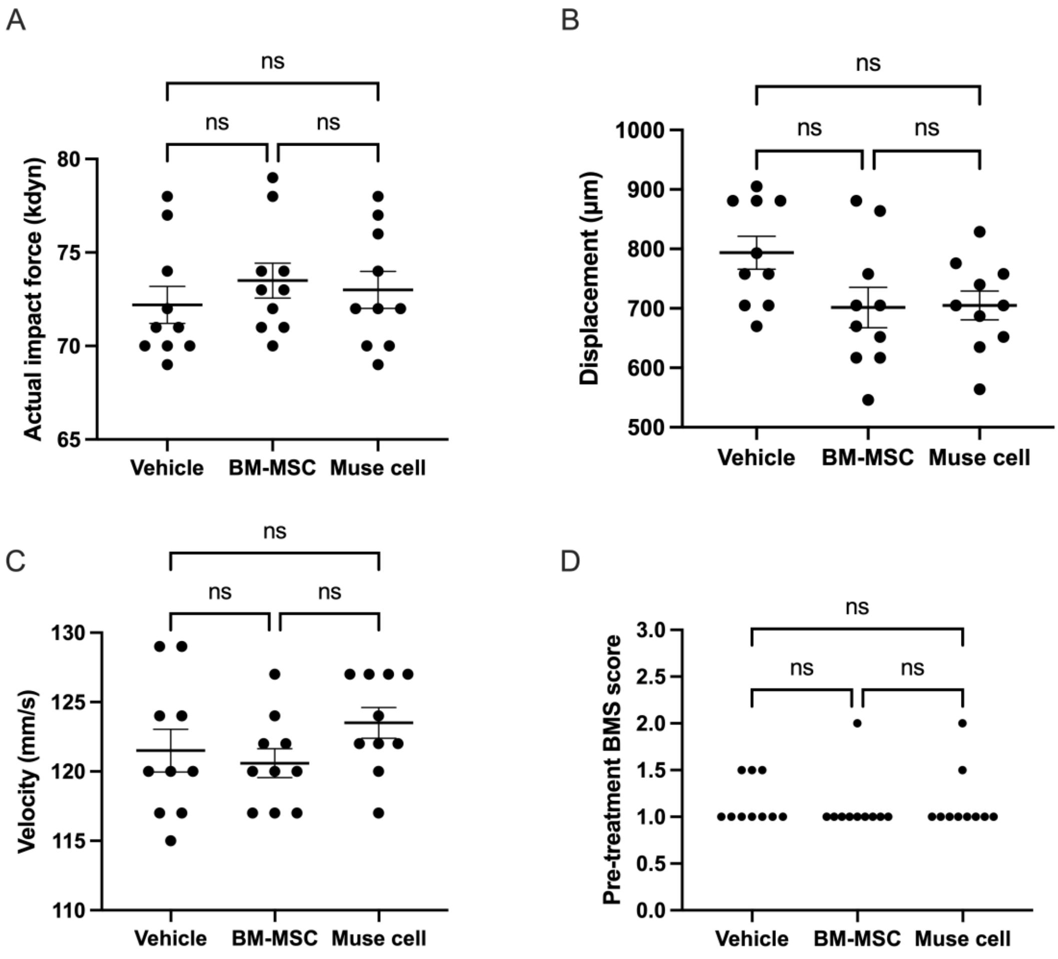

Figure S2. Baseline injury parameters and pre-treatment locomotor scores. (A) Actual impact force, (B) displacement, and (C) velocity during spinal cord contusion injury were comparable among the vehicle, BM-MSC, and Muse cell groups. (D) Pre-treatment Basso Mouse Scale (BMS) scores showed no significant differences among the groups. Data are presented as mean ± SEM. ns, not significant.

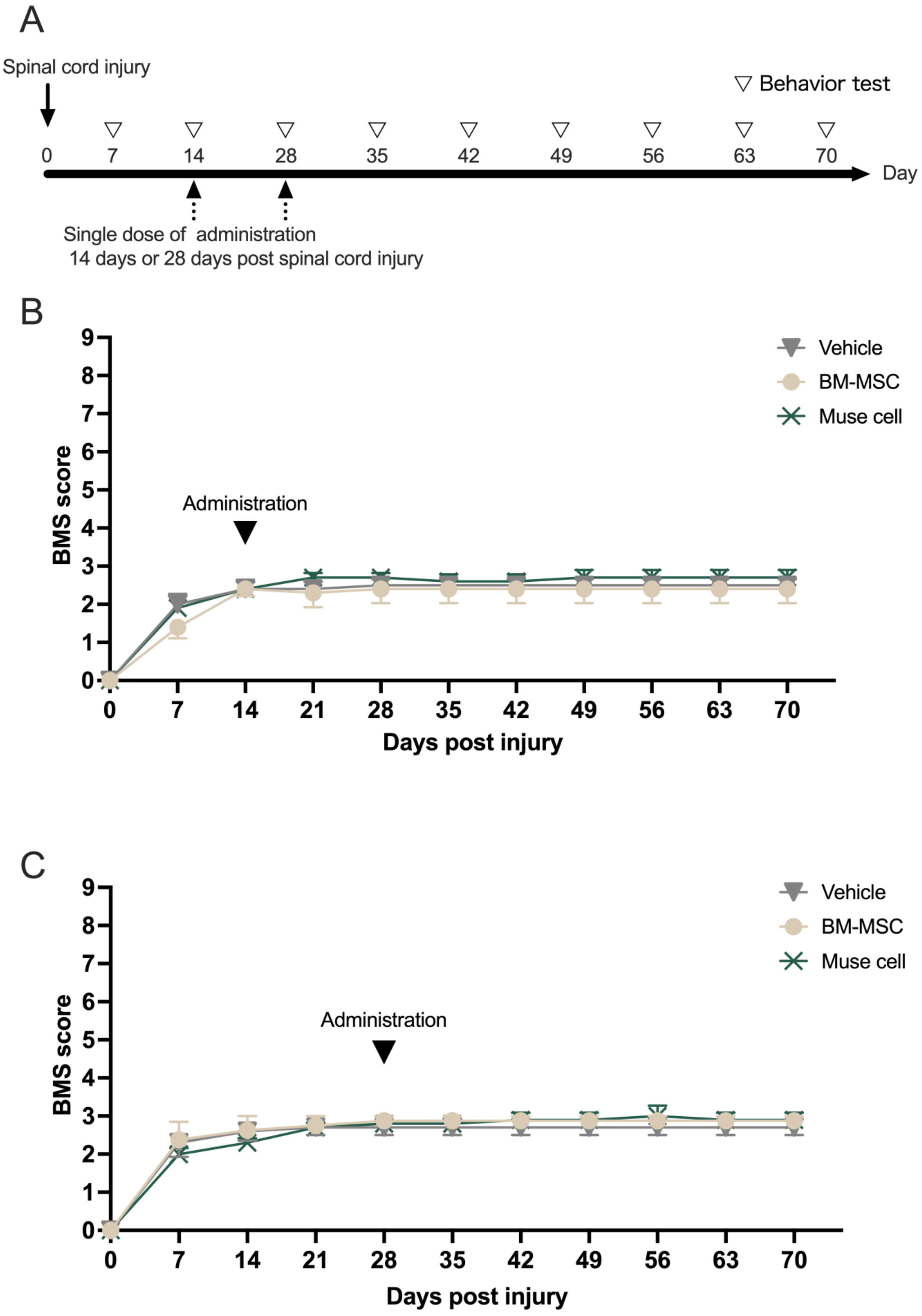
A
Spinal cord injury
▽ Behavior test
0
7
14
28
35
42
49
56
63
70
Day
Single dose of administration
14 days or 28 days post spinal cord injury
B
BMS score
Administration
Vehicle
BM-MSC
Muse cell
Days post injury
C
BMS score
Administration
Vehicle
BM-MSC
Muse cell
Days post injury

Figure S3 Behavioral outcomes in the 14- and 28-DPI treatment cohorts

(A) Experimental design. The 14- and 28-DPI treatment cohorts received a single tail-vein injection of human Muse cells (5×10^4 cells in 0.10 mL PBS), human BM-MSC (5×10^4 cells in 0.10 mL PBS), or vehicle (0.10 mL PBS). No immunosuppressants were used. Hindlimb locomotion was evaluated weekly with the BMS. Tissue was collected at 10 weeks post-injury. Interim BMS analyses showed no treatment-related recovery trend; enrollment was capped at $n = 5$ per treatment group.

(B) 14-DPI cohort ($n = 5$ per treatment group). No significant improvement in hindlimb function was observed among the three groups during the observation period.

(C) 28-DPI cohort ($n = 5$ per treatment group). No significant improvement in hindlimb function was observed among the three groups during the observation period.

Data are mean ± SEM.

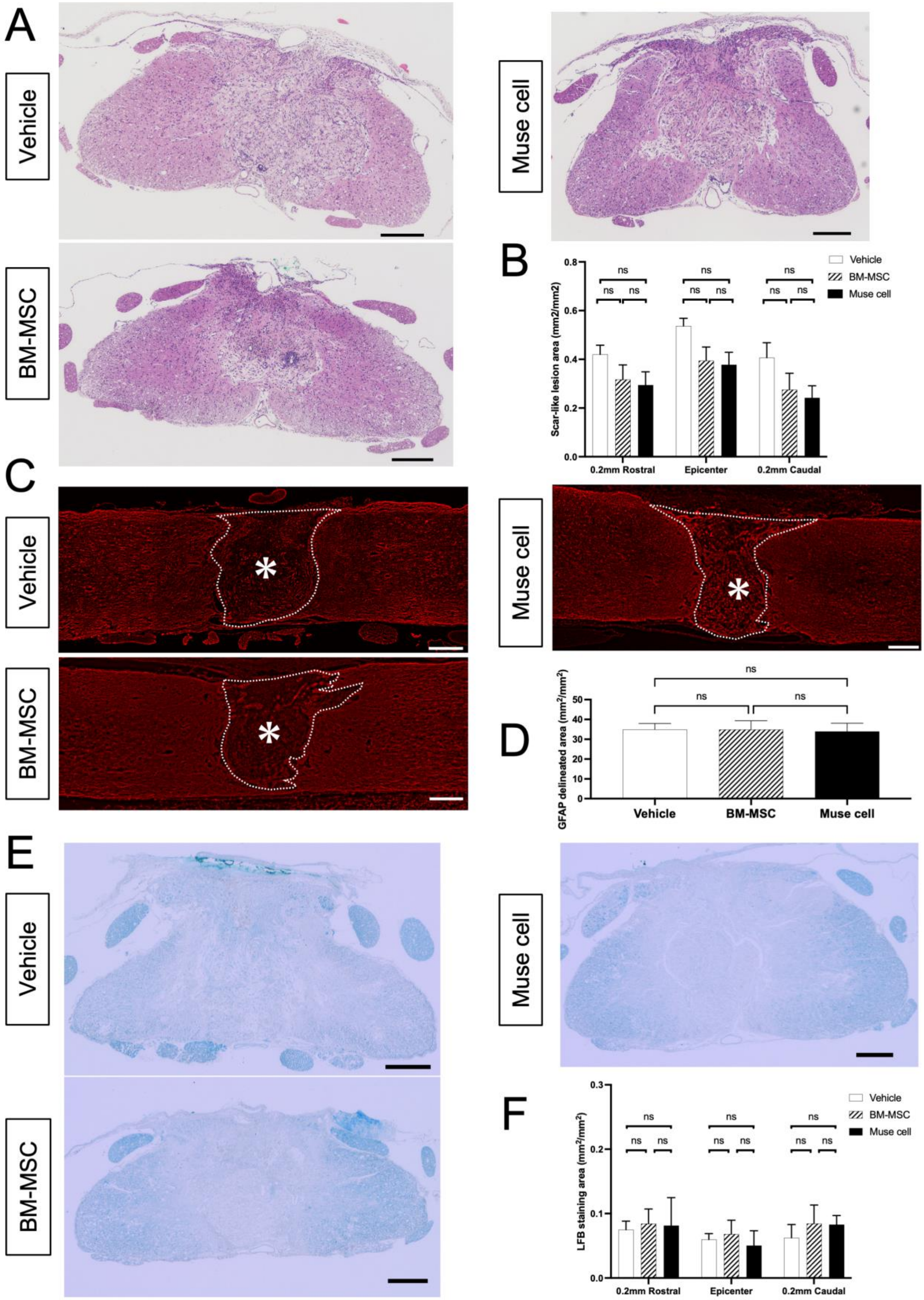

A
Vehicle
Muse cell
BM-MSC
B
Scar-like lesion area (mm2/mm2)
Vehicle
BM-MSC
Muse cell
ns
0.2mm Rostral
Epicenter
0.2mm Caudal
C
Vehicle
Muse cell
BM-MSC
D
GFAP delineated area (mm²/mm²)
ns
Vehicle
BM-MSC
Muse cell
E
Vehicle
Muse cell
BM-MSC
F
LFB staining area (mm²/mm²)
Vehicle
BM-MSC
Muse cell
ns
0.2mm Rostral
Epicenter
0.2mm Caudal

Figure S4 Histologic analysis (HE, GFAP, LFB) of spinal cord lesions in 8-DPI cohort at 42 days after spinal cord injury

(A) Representative HE staining at the lesion epicenter.

(B) Quantification of the scar-like lesion area (n = 5 per treatment group). No significant differences were observed among the three groups.

(C) Representative glial scar area.

(D) Quantification of glial scar area ($n = 5$ per treatment group). No significant differences were observed among the three groups.

(E) Representative LFB staining at the lesion epicenter.

(F) Spared myelin quantification ($n = 5$ per treatment group). No significant differences were observed among the three groups.

Data are mean ± SEM. Symbol: ns, not significant. Scale bars in A,C, and E = 200 μm.

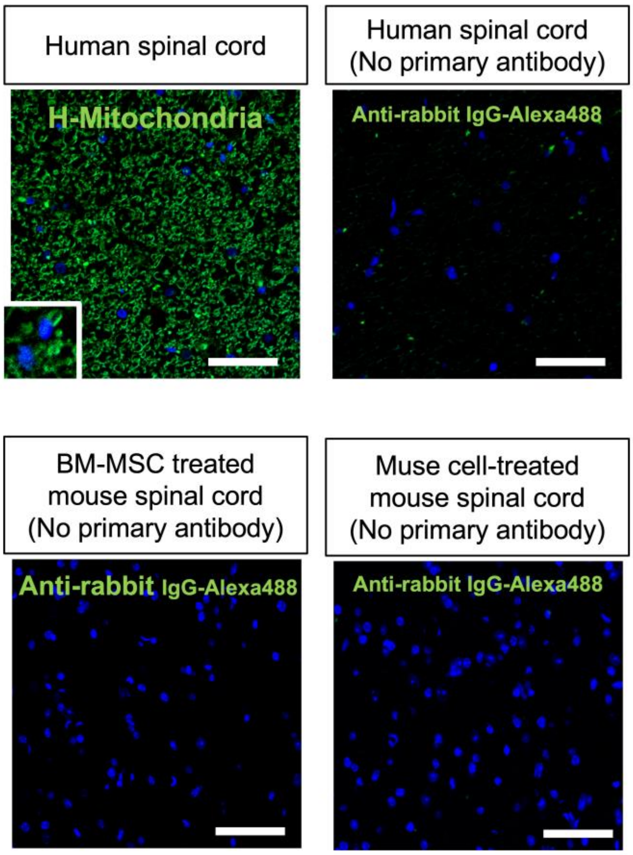

Figure S5 Specificity control for anti-human mitochondrial staining.

Human spinal cord tissue showed positive anti-human mitochondrial staining, whereas no-primary-antibody controls in human spinal cord, BM-MSC-treated mouse spinal cord, and Muse cell-treated mouse spinal cord showed minimal secondary antibody signal. Green indicates anti-human mitochondrial or anti-rabbit IgG-Alexa 488 signal; blue indicates DAPI. Scale bars = 50 μm.

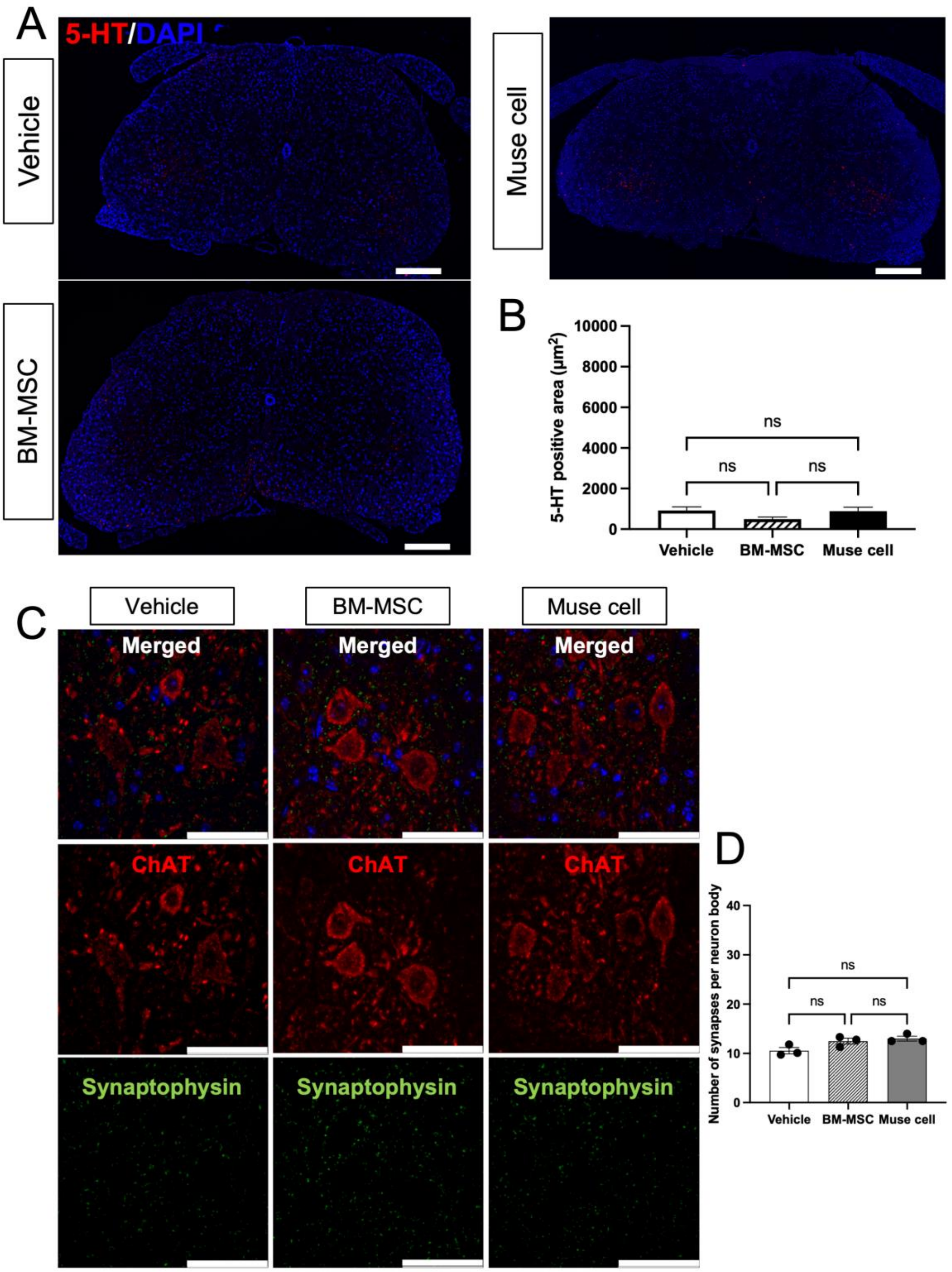

Figure S6 Lumbar enlargement in 8-DPI cohort at 42 days after spinal cord injury: 5-HT–positive area and perisomatic synapses

(A) Representative 5-HT immunofluorescence.

(B) 5-HT–positive area quantification ($n = 3$ per treatment group). No significant differences were observed among the three groups.

(C) Representative perisomatic synapses apposed to ChAT-positive motor neurons.

(D) Number of synapses per motor neuron ($n = 3$ per treatment group). No significant differences were observed among the three groups.

Data are mean ± SEM. Symbol: ns, not significant. Scale bars in (A) = 200 μm; scale bars in (C) = 50 μm.
